# Bis(3-amino­pyrazine-2-carboxyl­ato-κ^2^ *N* ^1^,*O*)diaqua­manganese(II)

**DOI:** 10.1107/S1600536810034835

**Published:** 2010-09-08

**Authors:** Min-Yan Zheng, Yong-Sheng Wei, Wei Geng, Guang Fan

**Affiliations:** a College of Chemistry & Chemical Engineering, Xianyang Normal University, Xianyang 712000, Shaanxi, People’s Republic of China

## Abstract

The Mn^II^ atom in the title compound, [Mn(C_5_H_4_N_3_O_2_)_2_(H_2_O)_2_], exhibits an octa­hedral geometry comprising the two O atoms and two N atoms from two 3-amino­pyrazine-2-carboxyl­ate ligands, which act as chelating ligands, and two water mol­ecules. An intra­molecular N—H⋯O hydrogen bond occurs. In the crystal, N—H⋯O, O—H⋯N and O—H⋯O hydrogen bonds link adjacent mol­ecules into a three-dimensional network. The mol­ecule lies on a twofold rotation axis.

## Related literature

For the nickel(II) analog, see: Ptasiewicz-Bak & Leciejewicz (1999 ▶).
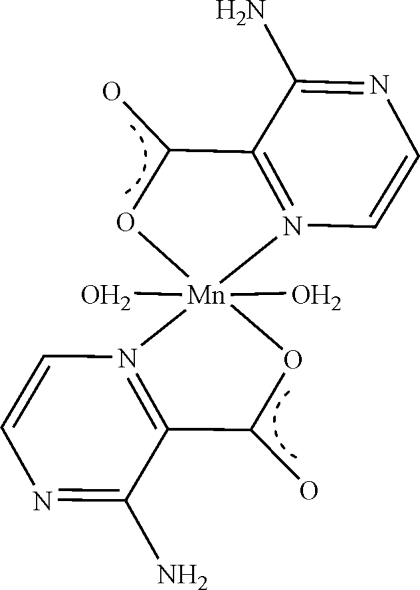


## Experimental

### 

#### Crystal data

[Mn(C_5_H_4_N_3_O_2_)_2_(H_2_O)_2_]
*M* *_r_* = 367.20
Monoclinic, 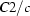

*a* = 7.9257 (11) Å
*b* = 12.6994 (18) Å
*c* = 13.663 (2) Å
β = 91.903 (2)°
*V* = 1374.4 (3) Å^3^
*Z* = 4
Mo *K*α radiation
μ = 1.01 mm^−1^
*T* = 296 K
0.12 × 0.10 × 0.08 mm

#### Data collection

Bruker SMART APEX diffractometer
Absorption correction: multi-scan (*SADABS*; Sheldrick, 1996 ▶) *T*_min_ = 0.889, *T*_max_ = 0.924
3373 measured reflections
1221 independent reflections
1114 reflections with *I* > 2σ(*I*)
*R*_int_ = 0.021

#### Refinement

*R*[*F* ^2^ > 2σ(*F* ^2^)] = 0.031
*wR*(*F* ^2^) = 0.096
*S* = 1.09
1221 reflections
112 parameters
2 restraints
H atoms treated by a mixture of independent and constrained refinement
Δρ_max_ = 0.36 e Å^−3^
Δρ_min_ = −0.22 e Å^−3^

### 

Data collection: *SMART* (Bruker, 2000 ▶); cell refinement: *SAINT* (Bruker, 2000 ▶); data reduction: *SAINT*; program(s) used to solve structure: *SHELXS97* (Sheldrick, 2008 ▶); program(s) used to refine structure: *SHELXL97* (Sheldrick, 2008 ▶); molecular graphics: *SHELXTL* (Sheldrick, 2008 ▶); software used to prepare material for publication: *SHELXL97*.

## Supplementary Material

Crystal structure: contains datablocks I, global. DOI: 10.1107/S1600536810034835/ng5015sup1.cif

Structure factors: contains datablocks I. DOI: 10.1107/S1600536810034835/ng5015Isup2.hkl

Additional supplementary materials:  crystallographic information; 3D view; checkCIF report

## Figures and Tables

**Table 1:** Hydrogen-bond geometry (Å, °)

*D*—H⋯*A*	*D*—H	H⋯*A*	*D*⋯*A*	*D*—H⋯*A*
N3—H3*C*⋯O1^i^	0.86	2.33	3.044 (3)	141
N3—H3*D*⋯O2	0.86	2.07	2.703 (4)	130
O3—H3*B*⋯N2^ii^	0.89 (1)	1.95 (1)	2.833 (3)	170 (3)
O3—H3*A*⋯O2^iii^	0.89 (1)	1.75 (1)	2.637 (3)	171 (3)

Symmetry codes: (i) 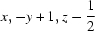; (ii) 
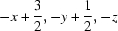
; (iii) 
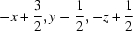
.

**Fig. 1.**
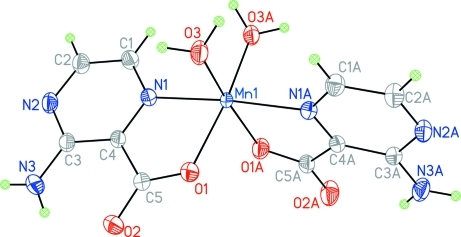
A view of the molecular structure with the atom-labling scheme. Displacement ellipsoids are drawn at the 30% probability level and H atoms are shown as small spheres of arbitrary radii.

**Fig. 2.**
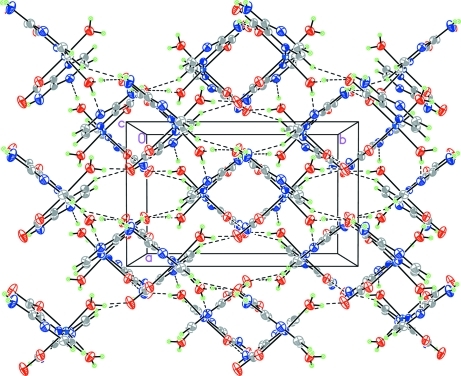
Three dimensional network of the title complex connected through hydrogen bonding.

## supplementary crystallographic information

### Experimental

The title complex was obtained as the main phase from the hydrothermal reaction of manganese sulfate tetrahydrate (0.0189 g) and 3-aminopyrazine-2-carboxylic acid (0.0913 g) in a 1:2 molar ratio. The reactants along with water were placed in a Teflon-lined stainless steel Parr bomb; the bomb was held at 413 K for three days. After cooling to room temperature, pink crystals were obtained.

### Refinement

All H atoms attached to C atoms and O atom from organic ligand were generated in idealized positions and constrained to ride on their parental C atoms, with C—H=0.93 Å, N—H=0.86 Å and and *U*_iso_(H) = 1.5*U*(C). The water H-atoms were located in a difference Fouier map, and were refined with a distance restraint of O–H 0.88+0.01 Å; their temperature factors were also tied to those of the O-atom.

### Crystal data

**Table:**

[Mn(C_5_H_4_N_3_O_2_)_2_(H_2_O)_2_]	*F*(000) = 748
*M**_r_* = 367.20	*D*_x_ = 1.775 Mg m^−^^3^
Monoclinic, *C*2/*c*	Mo *K*α radiation, λ = 0.71073 Å
*a* = 7.9257 (11) Å	Cell parameters from 117 reflections
*b* = 12.6994 (18) Å	θ = 2.5–18.9°
*c* = 13.663 (2) Å	µ = 1.01 mm^−^^1^
β = 91.903 (2)°	*T* = 296 K
*V* = 1374.4 (3) Å^3^	Block, pink
*Z* = 4	0.12 × 0.10 × 0.08 mm

### Data collection

**Table:**

Bruker SMART APEX diffractometer	1221 independent reflections
Radiation source: fine-focus sealed tube	1114 reflections with *I* > 2σ(*I*)
graphite	*R*_int_ = 0.021
φ and ω scans	θ_max_ = 25.1°, θ_min_ = 3.0°
Absorption correction: multi-scan (*SADABS*; Sheldrick, 1996)	*h* = −7→9
*T*_min_ = 0.889, *T*_max_ = 0.924	*k* = −15→15
3373 measured reflections	*l* = −16→12

### Refinement

**Table:**

Refinement on *F*^2^	Secondary atom site location: difference Fourier map
Least-squares matrix: full	Hydrogen site location: inferred from neighbouring sites
*R*[*F*^2^ > 2σ(*F*^2^)] = 0.031	H atoms treated by a mixture of independent and constrained refinement
*wR*(*F*^2^) = 0.096	*w* = 1/[σ^2^(*F*_o_^2^) + (0.0509*P*)^2^ + 1.9431*P*] where *P* = (*F*_o_^2^ + 2*F*_c_^2^)/3
*S* = 1.09	(Δ/σ)_max_ < 0.001
1221 reflections	Δρ_max_ = 0.36 e Å^−^^3^
112 parameters	Δρ_min_ = −0.22 e Å^−^^3^
2 restraints	Extinction correction: *SHELXL97* (Sheldrick, 2008), Fc^*^=kFc[1+0.001xFc^2^λ^3^/sin(2θ)]^-1/4^
Primary atom site location: structure-invariant direct methods	Extinction coefficient: 0.0048 (10)

### Special details

**Table:**

Geometry. All e.s.d.'s (except the e.s.d. in the dihedral angle between two l.s. planes) are estimated using the full covariance matrix. The cell e.s.d.'s are taken into account individually in the estimation of e.s.d.'s in distances, angles and torsion angles; correlations between e.s.d.'s in cell parameters are only used when they are defined by crystal symmetry. An approximate (isotropic) treatment of cell e.s.d.'s is used for estimating e.s.d.'s involving l.s. planes.
Refinement. Refinement of *F*^2^ against ALL reflections. The weighted *R*-factor *wR* and goodness of fit *S* are based on *F*^2^, conventional *R*-factors *R* are based on *F*, with *F* set to zero for negative *F*^2^. The threshold expression of *F*^2^ > σ(*F*^2^) is used only for calculating *R*-factors(gt) *etc*. and is not relevant to the choice of reflections for refinement. *R*-factors based on *F*^2^ are statistically about twice as large as those based on *F*, and *R*- factors based on ALL data will be even larger.

### Fractional atomic coordinates and isotropic or equivalent isotropic displacement parameters (Å^2^)

**Table:**

	*x*	*y*	*z*	*U*_iso_*/*U*_eq_	
Mn1	0.5000	0.30461 (4)	0.2500	0.0254 (2)	
O1	0.6806 (3)	0.42418 (16)	0.23675 (14)	0.0466 (5)	
O2	0.8132 (3)	0.52317 (18)	0.12895 (17)	0.0645 (7)	
O3	0.6893 (3)	0.19335 (16)	0.26050 (16)	0.0475 (5)	
N1	0.5308 (3)	0.31144 (16)	0.09621 (16)	0.0332 (5)	
N2	0.5990 (3)	0.3363 (2)	−0.09977 (17)	0.0425 (6)	
N3	0.7654 (4)	0.4795 (2)	−0.0636 (2)	0.0573 (8)	
H3C	0.7829	0.4858	−0.1251	0.069*	
H3D	0.8113	0.5231	−0.0225	0.069*	
C1	0.4598 (3)	0.2483 (2)	0.0281 (2)	0.0396 (7)	
H1	0.3855	0.1957	0.0463	0.048*	
C2	0.4971 (4)	0.2615 (2)	−0.0691 (2)	0.0430 (7)	
H2	0.4485	0.2158	−0.1151	0.052*	
C3	0.6674 (4)	0.4023 (2)	−0.0320 (2)	0.0386 (6)	
C4	0.6355 (3)	0.3870 (2)	0.0689 (2)	0.0342 (6)	
C5	0.7160 (4)	0.4498 (2)	0.1509 (2)	0.0406 (7)	
H3B	0.764 (3)	0.181 (2)	0.2150 (18)	0.049*	
H3A	0.678 (4)	0.1343 (15)	0.295 (2)	0.049*	

### Atomic displacement parameters (Å^2^)

**Table:**

	*U*^11^	*U*^22^	*U*^33^	*U*^12^	*U*^13^	*U*^23^
Mn1	0.0323 (3)	0.0239 (3)	0.0203 (3)	0.000	0.0081 (2)	0.000
O1	0.0624 (13)	0.0430 (11)	0.0349 (11)	−0.0126 (10)	0.0109 (9)	−0.0046 (9)
O2	0.0916 (18)	0.0529 (14)	0.0506 (14)	−0.0358 (13)	0.0258 (13)	−0.0123 (11)
O3	0.0556 (13)	0.0492 (13)	0.0389 (12)	0.0155 (10)	0.0185 (10)	0.0073 (9)
N1	0.0350 (12)	0.0326 (12)	0.0324 (12)	−0.0001 (9)	0.0070 (9)	0.0017 (9)
N2	0.0480 (14)	0.0488 (14)	0.0314 (12)	0.0033 (11)	0.0100 (10)	0.0017 (11)
N3	0.083 (2)	0.0478 (15)	0.0422 (15)	−0.0173 (14)	0.0232 (14)	0.0036 (12)
C1	0.0380 (15)	0.0445 (16)	0.0364 (15)	−0.0049 (12)	0.0022 (11)	−0.0001 (13)
C2	0.0429 (16)	0.0516 (17)	0.0347 (15)	−0.0040 (14)	0.0034 (12)	−0.0018 (13)
C3	0.0444 (16)	0.0358 (14)	0.0363 (15)	0.0057 (12)	0.0127 (12)	0.0038 (12)
C4	0.0380 (14)	0.0307 (13)	0.0346 (14)	0.0032 (11)	0.0113 (11)	0.0014 (11)
C5	0.0501 (17)	0.0339 (14)	0.0386 (16)	−0.0052 (13)	0.0162 (13)	−0.0027 (12)

### Geometric parameters (Å, °)

**Table:**

Mn1—O3^i^	2.062 (2)	N1—C1	1.337 (3)
Mn1—O3	2.062 (2)	N2—C2	1.324 (4)
Mn1—O1	2.099 (2)	N2—C3	1.350 (4)
Mn1—O1^i^	2.099 (2)	N3—C3	1.332 (4)
Mn1—N1	2.125 (2)	N3—H3C	0.8600
Mn1—N1^i^	2.125 (2)	N3—H3D	0.8600
O1—C5	1.257 (3)	C1—C2	1.381 (4)
O2—C5	1.252 (3)	C1—H1	0.9300
O3—H3B	0.887 (10)	C2—H2	0.9300
O3—H3A	0.891 (10)	C3—C4	1.422 (4)
N1—C4	1.330 (3)	C4—C5	1.501 (4)
			
O3^i^—Mn1—O3	93.50 (13)	C1—N1—Mn1	127.13 (18)
O3^i^—Mn1—O1	170.52 (8)	C2—N2—C3	117.6 (2)
O3—Mn1—O1	90.29 (9)	C3—N3—H3C	120.0
O3^i^—Mn1—O1^i^	90.29 (9)	C3—N3—H3D	120.0
O3—Mn1—O1^i^	170.52 (8)	H3C—N3—H3D	120.0
O1—Mn1—O1^i^	87.32 (12)	N1—C1—C2	119.9 (3)
O3^i^—Mn1—N1	93.80 (8)	N1—C1—H1	120.1
O3—Mn1—N1	89.40 (8)	C2—C1—H1	120.1
O1—Mn1—N1	77.54 (8)	N2—C2—C1	123.0 (3)
O1^i^—Mn1—N1	99.02 (8)	N2—C2—H2	118.5
O3^i^—Mn1—N1^i^	89.40 (8)	C1—C2—H2	118.5
O3—Mn1—N1^i^	93.80 (8)	N3—C3—N2	117.4 (3)
O1—Mn1—N1^i^	99.02 (8)	N3—C3—C4	122.6 (3)
O1^i^—Mn1—N1^i^	77.54 (8)	N2—C3—C4	120.0 (3)
N1—Mn1—N1^i^	175.33 (11)	N1—C4—C3	120.2 (2)
C5—O1—Mn1	116.20 (18)	N1—C4—C5	115.3 (2)
Mn1—O3—H3B	125 (2)	C3—C4—C5	124.5 (2)
Mn1—O3—H3A	122 (2)	O2—C5—O1	125.1 (3)
H3B—O3—H3A	107 (3)	O2—C5—C4	117.8 (2)
C4—N1—C1	119.3 (2)	O1—C5—C4	117.2 (2)
C4—N1—Mn1	113.60 (17)		

Symmetry codes: (i) −*x*+1, *y*, −*z*+1/2.

### Hydrogen-bond geometry (Å, °)

**Table:**

*D*—H···*A*	*D*—H	H···*A*	*D*···*A*	*D*—H···*A*
N3—H3C···O1^ii^	0.86	2.33	3.044 (3)	141
N3—H3D···O2	0.86	2.07	2.703 (4)	130
O3—H3B···N2^iii^	0.89 (1)	1.95 (1)	2.833 (3)	170 (3)
O3—H3A···O2^iv^	0.89 (1)	1.75 (1)	2.637 (3)	171 (3)

Symmetry codes: (ii) *x*, −*y*+1, *z*−1/2; (iii) −*x*+3/2, −*y*+1/2, −*z*; (iv) −*x*+3/2, *y*−1/2, −*z*+1/2.
